# 18S rDNA Phylogeny of *Lamproderma* and Allied Genera (Stemonitales, Myxomycetes, Amoebozoa)

**DOI:** 10.1371/journal.pone.0035359

**Published:** 2012-04-18

**Authors:** Anna Maria Fiore-Donno, Akiko Kamono, Marianne Meyer, Martin Schnittler, Manabu Fukui, Thomas Cavalier-Smith

**Affiliations:** 1 Institute of Botany and Landscape Ecology, University of Greifswald, Greifswald, Germany; 2 The Institute of Low Temperature Science, Hokkaido University, Sapporo, Japan; 3 Le Bayet, Rognaix, France; 4 Zoology Department, University of Oxford, Oxford, United Kingdom; University of Florida, United States of America

## Abstract

The phylogenetic position of the slime-mould genus *Lamproderma* (Myxomycetes, Amoebozoa) challenges traditional taxonomy: although it displays the typical characters of the order Stemonitales, it appears to be sister to Physarales. This study provides a small subunit (18S or SSU) ribosomal RNA gene-based phylogeny of *Lamproderma* and its allies, with new sequences from 49 specimens in 12 genera. We found that the order Stemonitales and *Lamproderma* were both ancestral to Physarales and that *Lamproderma* constitutes several clades intermingled with species of *Diacheopsis*, *Colloderma* and *Elaeomyxa*. We suggest that these genera may have evolved from *Lamproderma* by multiple losses of fruiting body stalks and that many taxonomic revisions are needed. We found such high genetic diversity within three *Lamproderma* species that they probably consist of clusters of sibling species. We discuss the contrasts between genetic and morphological divergence and implications for the morphospecies concept, highlighting the phylogenetically most reliable morphological characters and pointing to others that have been overestimated. In addition, we showed that the first part (∼600 bases) of the SSU rDNA gene is a valuable tool for phylogeny in Myxomycetes, since it displayed sufficient variability to distinguish closely related taxa and never failed to cluster together specimens considered of the same species.

## Introduction

Myxomycetes or plasmodial slime-moulds are distinctive amoebae that form macroscopic fruiting bodies, very common on litter or decaying logs in forests. These protists form a monophyletic taxon in the phylum Amoebozoa [1], [2]. Myxomycetes include more species (about 900 recognized) [3] than all other Amoebozoa [4]; they are present in nearly every terrestrial environment but also, as amoebae or flagellates, in aquatic environments where they cannot form fruiting bodies [5], [6], [7], [8], [9]. They have a complex life cycle culminating in the formation of mainly macroscopic fruiting bodies highly variable in shape and colour. Recently, it has been shown that they are one of the major components of the soil community [10]. Their unexpectedly high abundance in soil DNA samples suggests that currently available data on the distribution of Myxomycetes are likely to be incomplete, as non-fruiting stages are not taken into account.

Currently virtually all myxomycete species are based on morphological characters only. Though such a morphospecies concept was the only practical one prior to DNA sequencing, it has drawbacks. Firstly, available characters are rather few and it is difficult to judge their relative importance. Secondly, genetic studies have demonstrated that morphospecies sometimes lump together several biological species [11]. Thirdly, descriptions based only on one or a few specimens could lead to aberrant forms being wrongly established as species [12]; in the past some taxa have been described using unstable characters, perhaps influenced by environmental conditions (e.g. *Lamproderma granulosum*) [13]. Despite these drawbacks, there is currently no alternative to the morphospecies concept for conducting large-scale inventories and ecological studies.

Myxomycetes are classically described as having a complex life-cycle involving a sexual diploid stage, the multinucleate plasmodium, which grows from a zygote formed by fusion of two amoebae [14]. What is known of myxomycete genetics stems mostly from studies conducted from the sixties to the nineties [15], [16], based on tests of interbreeding compatibility between strains, which are limited to the minority of species that can be isolated and cultured in the laboratory. Nevertheless, the picture that emerged from these pioneering studies was that each morphospecies may be composed of an intricate pattern of core sexual strains and swarms of asexually reproducing clones that are related to them but differ genetically and may also differ in observable morphological traits [17]. Recently, this assumption was tested by assessing the genetic variability in natural populations of two *Lamproderma* species. Both species were composed of several discrete lineages and each lineage consisted of sequences identical for the small subunit rRNA (SSU) gene, the internal transcribed spacer (ITS1) that lies between the SSU and the 5.8S rRNA gene and an intron of the elongation factor EF-1α [13], [18]. This is explained best by an asexual mode of reproduction, challenging the classical view, although the existence of cryptic species cannot be excluded. The morphospecies concept thus gives an oversimplified picture of myxomycete species and further study of their genetic variation is necessary to clarify their population biology, evolution, and species boundaries.

**Figure 1:**
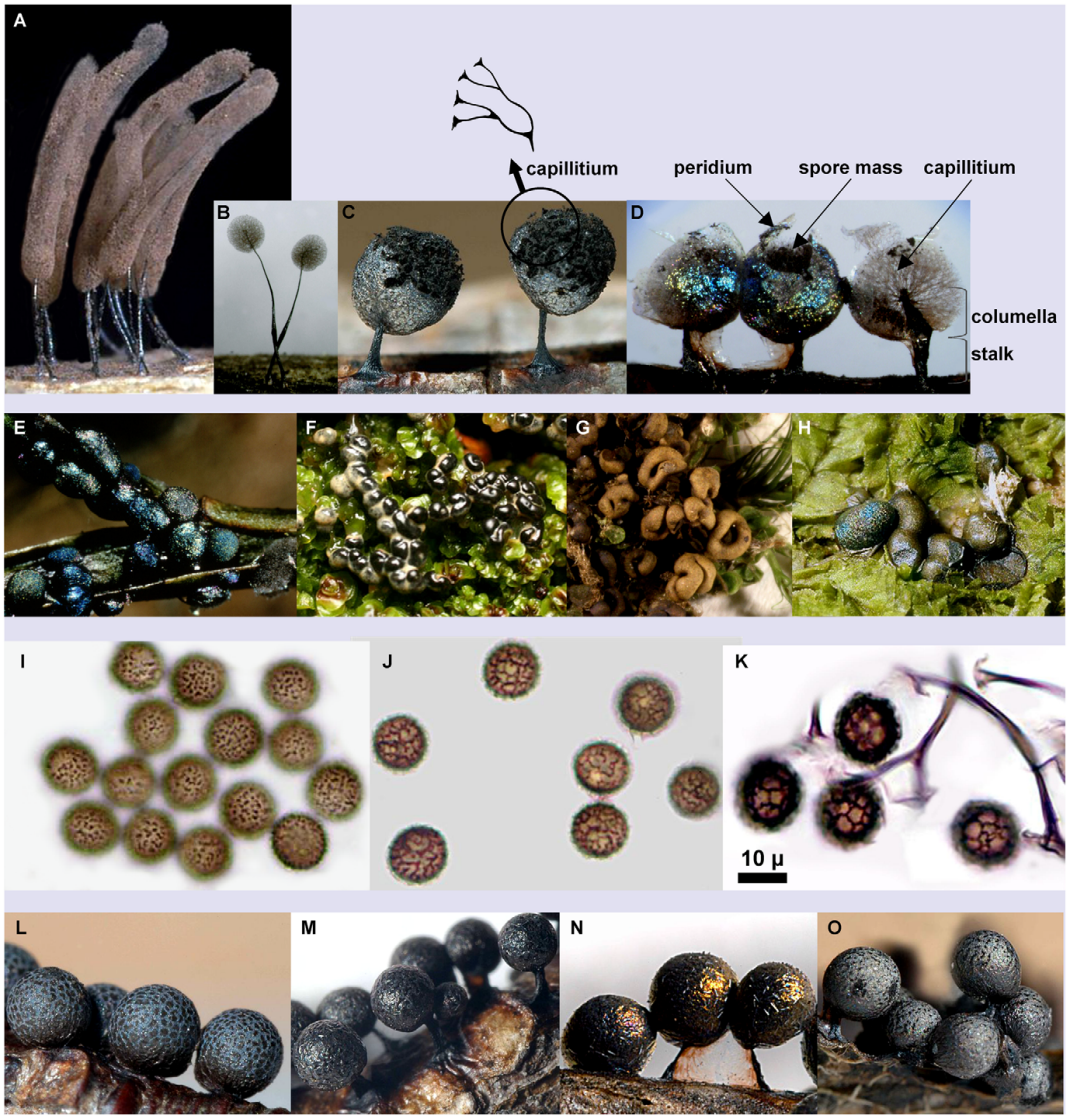
Photos of Stemonitales. **A–D: Peridium types in Stemonitales;** A: Early evanescent peridium: *Stemonitis pallida* ^1^; B: Early evanescent peridium: *Comatricha nigra* ^1^; C: Splitting out peridium: *Meriderma spinulosporum* ^1^; D: Persistent, shining peridium: *Lamproderma sauteri* ^2^. **E–H: Genera allied to** ***Lamproderma*** **;** E: *Diacheopsis metallica* ^2^; F: *Colloderma robustum*, developing sporophore^3^; G: *Colloderma robustum* ^4^; H: *Colloderma oculatum* ^1^. **I–K: Spore ornamentation in** ***Meriderma*** **;** I: Spinulose in *M. echinulatum* ^1^; J: Nearly complete reticulum in *M. carestiae* ^1^; K: Reticulate in *M. cribrarioides* ^1^. **L–O: The “** ***Lamproderma maculatum*** **" group;** L: *L. acanthosporum* ^1^; M: *L. maculatum* ^1^; N: *L. pseudomaculatum* (M–N: also with white splinters in the peridium)^1^; O: *L. echinosporum* ^1^. *(* ***Photos*** *: 1: Alain Michaud, 2: Michel Poulain, 3: Martin Schnittler, 4: Anna Maria Fiore-Donno)*.

We focus here on the genus *Lamproderma*, which has been intensively studied in the last two decades with many newly described morphospecies [19], [20], [21], and the order Stemonitales in which *Lamproderma* is currently classified. Stemonitales and Physarales differ in several ways. Physarales are characterized by the presence of lime deposits, absent in Stemonitales, and each order produces a distinct kind of melanin [22]. There are also developmental differences, e.g. the stalk is secreted inside the cytoplasm in Stemonitales [23] but in Physarales it is merely a constriction of the cytoplasm and its surrounding membrane [24]. Molecular studies based on a restricted sampling initially showed the dark-spored orders as two sister-clades [25], [26]. Surprisingly, when more taxa were added, phylogenies revealed Stemonitales as paraphyletic to Physarales [6], with *Lamproderma* a sister group to Physarales [27]. A key objective of the present study was to test whether this apparent paraphyly is stable to more extensive taxon sampling, especially of *Lamproderma* and other Stemonitales such as *Comatricha*. Past monographs of the genus *Lamproderma* focused on characters that could not provide clear boundaries between *Comatricha* and *Lamproderma* [28], [29], [30]. A recent molecular phylogeny has shown that the two genera were clearly separated. A morphological character that provides easy distinction is the peridium (the sheath enveloping the spore mass) (Fig. 1). Three main types of peridium are found in dark-spored Myxomycetes: early evanescent in *Comatricha* and *Stemonitis*; early evanescent but splitting out in fragments in the *Lamproderma atrosporum* complex ( = new genus *Meriderma* [20]) and persistent in *Lamproderma* and Physarales [27] (Fig. 1 A–D).

*Lamproderma* includes species found at the edge of melting snow banks in spring . called nivicolous Myxomycetes [31], which have very narrow ecological requirements, fruiting in high-latitude or -altitude grasslands and forests [21], [32]. Under proper ecological conditions, they can cover several square metres of dead vegetation with their highly decorated fruiting bodies (Fig. 1). Other species of *Lamproderma*, termed bryophilous, can also be found in very damp ravines on substrates covered with mosses [33]. According to the most recent treatise, the genus comprises 50 species, 25 of which are nivicolous, four bryophilous (including the dubious *L. granulosum*) and the remaining 21 found on many different substrates (mostly dead wood and litter, but also living plants) [20].

**Figure 2:**
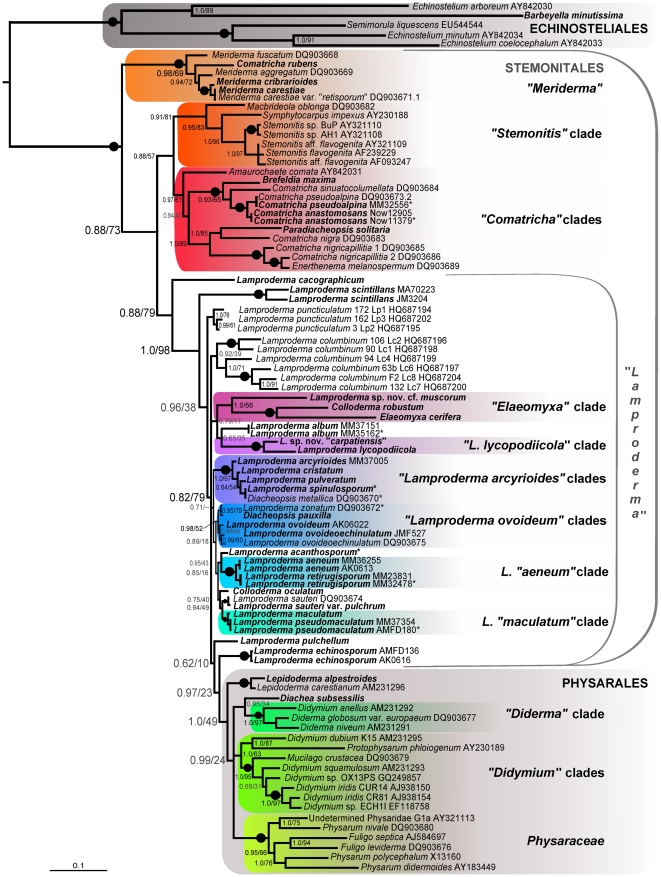
SSU rDNA gene tree of selected Stemonitales and Physarales derived by Bayesian inference of 1566 nucleotide positions of 84 taxa. Species names are followed by GenBank accession number, except for sequences obtained during this study (in bold), whose accession numbers and collection sites are in Table S1; sequences are marked by an asterisk if partial. Clades including more than one species are highlighted by a rectangle and arbitrarily named. Names of orders are in capitals (in grey if paraphyletic). Bayesian posterior probabilities (BPP)/ML bootstrap replicates (MLB) are shown for each branch, a dot on the line indicates maximum support in both analyses. Grey numbers indicate low support (BPP<0.9 or MLB<70%), dashes indicate a conflicting topology in the ML tree. The scale bar indicates the fraction of substitutions per site.

To investigate the relationship between genetic and morphological divergence we have now obtained 36 nearly complete and 13 partial new SSU rDNA gene sequences from *Lamproderma* and eleven allied genera (Table S1). The goals of this study are to: 1) provide tools for species delimitation in *Lamproderma*; 2) investigate *Lamproderma* phylogeny and relationships between *Lamproderma* and related genera; 3) elucidate the deep phylogeny of Stemonitales; 4) clarify the relationship between Stemonitales and Physarales, the two orders of dark-spored Myxomycetes.

## Materials and Methods

### Specimens

All specimens were field-collected and kept in herbaria (Table S1). To ensure a coherent approach for this taxonomically difficult group, all specimens were identified by the third author.

### Nomenclatural choice

Two nomenclatural codes have been applied to this group: for historical reasons most manuals and treatises refer to “Myxomycetes" and apply the Botanical Code, whilst the less frequent “Myxogastria" or “Myxogastrea" [1] and the Zoological Code are used by those who wish to emphasize that they are Protozoa, not Fungi (e.g. [14]). The present study used the Botanical Code for easier comparison with the most used identification books [20], [34], [35], [36], [37]. Specimens were named according to the most recent treatise [20], taxa not yet described being written between inverted commas.

### DNA extraction, amplification and sequencing

DNA was extracted from 5–6 adjacent sporophores (most probably arising from a single plasmodium) using the DNeasy plant mini-kit (Qiagen, Hilden, Germany). Sporophores were cooled to −80°C in a 2 ml safe-lock Eppendorf tube with a single metallic bead of 5 mm diameter and then disrupted using a ball mill (Retsch MM301, 1 min, 30 Hz). We followed the manufacturer's protocol except for the final step where DNA was eluted in 50 µl of the elution buffer (instead of 200 µl). Even so, the DNA was undetectable neither by electrophoresis or spectrophotometry. The presence of numerous and large introns, sometimes with strong secondary structure, required the use of the “primer walking" method. We used, along with primers already published [27], many primers matching only one sequence, often in the introns themselves (Fig. S1). Amplification parameters were adapted accordingly: elongation time depending on the length of the expected product (1–2 min) and annealing temperature according to the primer sequences (52–58°C). For samples whose sequences could not be completed because of large introns, we extracted RNA from 5–6 sporophores using SurePrep Plant/Fungi Total RNA Purification Kit (Fisher BioReagents). The whole SSU was then reverse transcribed using BioScript™ One-Step RT-PCR Kit (Bioline) and sequenced. Amplicons were purified using SureClean (Bioline) or with the PCR DNA and Gel Band Purification kit (GE Healthcare Life Science), then sequenced at the DNA sequencing facility at the University of Oxford.

### Alignments and secondary structure prediction

We obtained 35 nearly complete (1684–2339 bp excluding introns) and 14 partial (the first ca. 600 nucleotides) new SSU rRNA gene sequences (Table S1). We also completed and updated sequences DQ903671 and DQ903673. We assembled the new sequences with those of selected representatives of Stemonitales, Physarales and Echinosteliales, the latter as outgroup, according to current phylogenies of Myxomycetes [6], [25], [27]. Sequences retrieved from GenBank that were (nearly) identical between themselves or partial sequences in clades already well represented were not included. Sequences were aligned by hand using BioEdit version 7.0.9 [38]. Since the SSU displays an unusually high rate of evolution in Myxomycetes, particular attention was given to the secondary structure of the variable helices to detect sequence signatures to characterize taxa. The secondary structure of the variable helices was inferred using *Physarum polycephalum* as a template [39]. To elucidate the folding pattern of secondary structure elements the RNAalifold web server was used (http://rna.tbi.univie.ac.at/cgi-bin/RNAalifold.cgi, last accessed July 2011). Three masks were created, of 1404, 1490 and 1533 positions, and tested using PhyML v2.4.4 (100 non-parametric bootstraps under the model GTR+I+gamma with 8 categories) [40]. The most stringent mask excluded all variable helices, while in the two other increasing parts of the beginning and the end of the helices were kept. Ninety-four positions of the echinostelid sequences could not be aligned. The alignment of 84 taxa and three masks is available (Supporting Information S1). From this alignment we excluded four taxa that gave very long and unstable branches on the tree likely to confuse the analyses: *Stemonitopsis hyperopta, Stemonitopsis typhina, Lamproderma disseminatum* and *Lamproderma arcyrionema* (Table S1).

### Phylogenetic analyses

The general time reversible model taking into account a gamma-distributed rate heterogeneity among sites (GTR+I+gamma) [41], [42] was selected using jModelTest 0.1.1 [40], [42] under the Akaike Information Criterion (-lnL = 27486.3027; p-inv = 0.1050; gamma shape = 0.3420). Maximum likelihood (ML) analyses were run using RAxML 7.0.4 [43] with the GTR model of substitution and a 25 rate category discrete gamma distribution. The alignment comprised 896 patterns where the proportion of gaps and ambiguous nucleotides was negligible (0.03%). The best-scoring ML tree was inferred from 200 randomized starting Maximum Parsimony trees using the GTRMIX model (best log likelihood = −26037.218600). The best-scoring tree was used to report the confidence values as percentages obtained through 1000 non-parametric bootstraps under the GTRCAT model.

Bayesian search of tree space used MrBayes, version 3.1.2 [44] with the GTR+I+gamma model of substitution, the gamma distribution being approximated by eight categories. Two runs starting from different random trees were performed and sampled every ten generations, with eight simultaneous chains, for one million generations. Convergence and other similarity diagnostics were calculated every 5,000 generations and the first 25% trees of the cold chain were discarded as burnin (default settings of the program). Convergence of the two runs, evaluated by a standard deviation of split frequencies <0.01, was not reached after 2 million generations; therefore the swapping betweens runs was doubled (nswaps = 4) and the “temperature" lowered to 0.1. The sample frequency was reset to the default value (100). With these settings, 3 millions generations were run. Although a standard deviation of split frequencies <0.01 was not reached, the number of exchanges between chains were sufficient (min 0.36, max 0.47), and the convergence diagnostics PSRF were all <1.015. The log likelihoods of the trees summarized from the two runs were similar (−27543.73 and −27548.86), and the topologies were identical, differing only in posterior probabilities. The burnin was set to10,000, and the remaining 40,000 trees were summarized. The resulting log likelihood of the consensus tree was −27554.40 (alpha = 0.391844, pinvar = 0.147379) (Fig. 2).

To further investigate internal relationships of the paraphyletic “*Lamproderma*" (as outlined in Fig. 2) we made an alignment of these 46 taxa only, where most variable helices could be kept, totalling 1695 positions. RAxML and Bayesian analyses were conducted as described above. The main groups (as in Fig. 2) were recovered, but their mutual relationships were not better resolved (results not shown).

An alignment of the first part of the SSU only was used for seven taxa of the *L. arcyrioides* group (549 positions, no positions excluded but ends trimmed) with the addition of the partial sequences of *L. violaceum* (a non-nivicolous species) and *L.* cf. *arcyrioides* AMFD338 (collected in Japan; differing from the type by a peridium lacking white splinters). Only 20 positions were variable. The best model (GTR+G) was selected as above, approximated by four categories (log likelihood: −897.74). Using this model, the Bayesian analysis had two runs starting from random trees, with eight simultaneous chains; trees were sampled every ten generations for one million generations. The two runs converged nearly immediately; only the first 1000 trees were discarded (log likelihood = −906.13, alpha = 96.238004). The resulting tree was rooted according to the topology in Fig. 2.

## Results

### Phylogenetic analyses

The trees obtained with the three alignments (1404, 1497 and 1533 nt) were compared. The tree from the longest alignment showed the best support for the basal branches. The tree from the shortest alignment not only had lower basal supports, but also failed to retrieve a monophyletic Physarales. Therefore, the 1533 position alignment was selected for in-depth analyses. The tree, rooted with Echinosteliales, showed a monophyletic dark-spored clade (maximum support in all analyses), with Stemonitales paraphyletic to a monophyletic Physarales (Bayesian posterior probabilities (BPP): 1.0; ML bootstrap replicates (MLB): 49). The main clades previously recovered [27] were here confirmed: “*Meriderma*", “*Stemonitis*" and “*Comatricha*", while the monophyly of “*Lamproderma*" (including allied taxa) was never retrieved (Fig. 2).

In Physarales, Didymiaceae (*Diachea*, *Didymium*, *Diderma*, *Lepidoderma*, *Mucilago* and *Protophysarum*) were paraphyletic or in an unresolved position relative to a monophyletic Physaraceae (BPP: 1.0; MLB: 100). Three reproducible clades (i.e. retrieved in all analyses) stood at the base of Stemonitales: “*Stemonitis*" (0.91; 81), “*Comatricha*" (0.97; 61) and the newly erected genus *Meriderma* (1.0; 100). “*Meriderma*" sometimes appeared as sister to “*Lamproderma*", instead of in the basal position as in Fig. 2.

**Figure 3:**
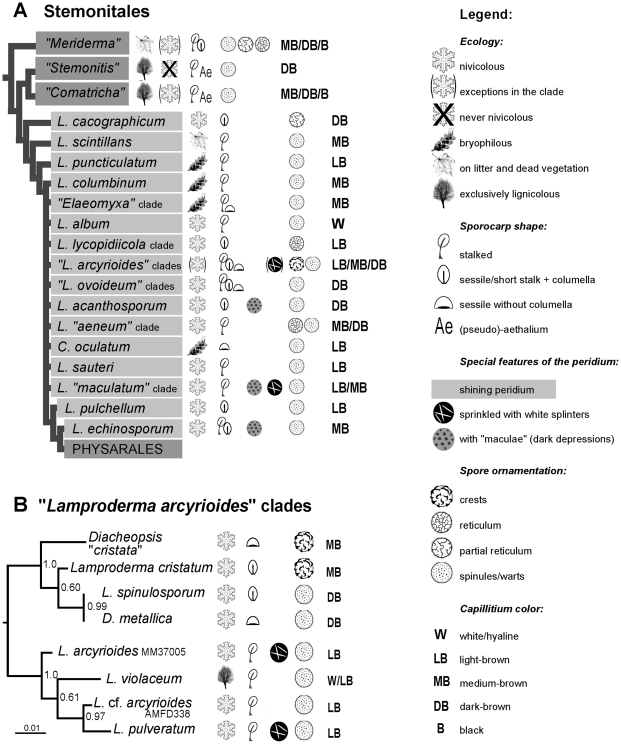
Relations between phylogeny and some morphological characters. **A:** Diagram of the major clades as in Fig. 2. Symbols refer only to taxa included in this study. **B:** Partial SSU gene tree of the “*Lamproderma arcyrioides*" clades derived by Bayesian inference of 549 nucleotide positions.

“*Lamproderma*" appeared as composed of diverging single-species branches (*L. cacographicum*, *L. pulchellum*) or clades of genetically different versions of single nominal *Lamproderma* species (*L. scintillans*, *L. puncticulatum*, *L. columbinum*, *L. echinosporum*), or clades grouping several *Lamproderma* species, or clades including *Lamproderma* and other genera (highlighted and named in Fig. 2). Although some of these clades were well supported (*L. scintillans*, *“L. arcyrioides"* clades), their reciprocal relationships were generally neither robust nor well supported. In particular, the position of the following taxa changed between alignments: *Lamproderma disseminatum* and *L. arcyrionema* (excluded from the final tree), *L. cacographicum*, *L. scintillans*, *L. pulchellum*, *L. acanthosporum* and *L. album*. Nevertheless, the basal position of *L. cacographicum*, *L. scintillans* and *L. puncticulatum* was reproducible, as was the position of *L. echinosporum* as sister to Physarales (Fig. 2). To facilitate comparison between phylogeny and ecological and morphological characters, Figure 3A shows selected ecological and morphological features for the main clades. Species fruiting exclusively on wood belonged to the clades “*Stemonitis*" and “*Comatricha*", but bryophilous and nivicolous species failed to cluster. Closely related nivicolous and non-nivicolous species were seen in “*Meriderma*" (all nivicolous except *Comatricha rubens*) and in “*Comatricha*" (nivicolous: *Comatricha pseudoalpina* and *C. sinuatocolumellata*, *C. anastomosans*, *C. nigricapillitia* and *Enerthenema melanospermum*) (Fig. 3A). A partial SSU sequence was obtained from a second specimen of the following taxa: *Comatricha anastomosans, C. pseudoalpina, Lamproderma aeneum, L. album, L. pseudomaculatum, L. retirugisporum, Meriderma carestiae, Stemonitopsis typhina* (Table S1). The partial sequences confirmed the phylogenetic assignment of the taxon in all cases: they were identical or displayed only few differences in the variable helices. Partial sequences that were identical to complete ones (*Comatricha anastomosans, C. pseudoalpina, Lamproderma pseudomaculatum, L. spinulosporum, Meriderma carestiae*) were excluded from the analysis to speed it but have been added to the tree in Fig. 2. The analysis of the seven taxa of the *L. arcyrioides* group indicates two subclades, one comprising *Diacheopsis “cristata"*, *L. cristatum* and *L. spinulosporum*/*Diacheopsis metallica* (the latter two identical despite different names) and the other the remaining four sequences (Fig. 3B).

Species determination and molecular phylogeny were concordant, since distinct sequences from a morphospecies always formed a clade (Fig. 2).

### Secondary structure prediction and search for signatures

**Figure 4:**
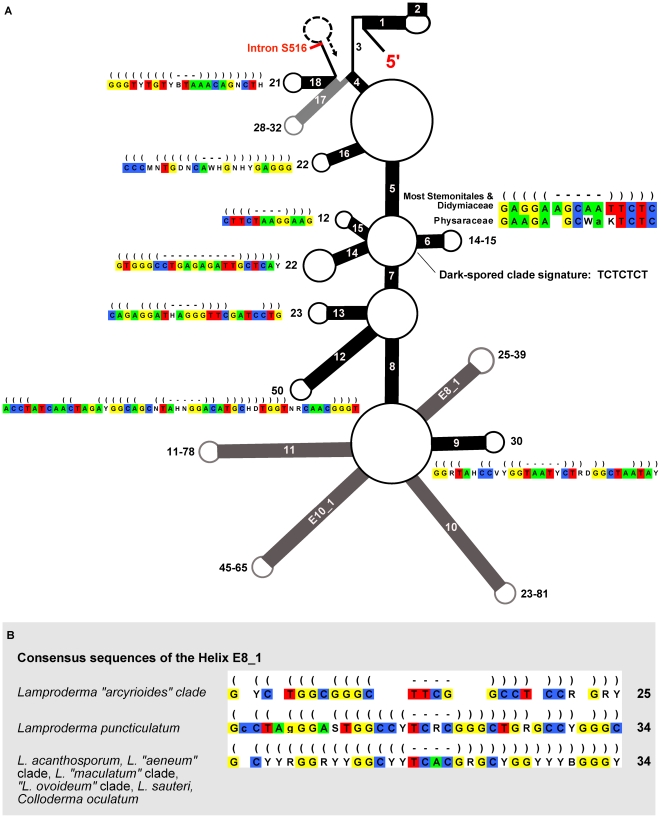
Secondary structure and signatures. **A:** Schematic secondary structure of the first part of the SSU rRNA (after [51]), encompassing the first 18 helices and part of the 19^th^, up to the first intron insertion site. Helices (paired strands) common to all domains of life are represented by black rectangles and numbered (E8_1, E10_1 are unique to eukaryotes), lengths are given next to the loops. Single-stranded segments are indicated by thin lines. For helices whose length is conserved, a 95% consensus is shown. Helices whose length is variable are in grey and the length range is given. Consensus sequences of the helices are shown in the Stockholm format (parentheses = stem, hyphens = loop, blank = no paring); lower-case characters indicate a base that is not present in all sequences of the group. **B:** Consensus sequences of the helix E8_1 of several *Lamproderma* clades, showing high variability and clade-specific sequence signatures. Lengths are more conservative than sequences.

We found great variation in the length of the helices of the first ∼600 bp of the SSU, in particular helices E8_1, 10, E10_1 and 11 (names according to [45] (Fig. 4A). To avoid ambiguities of alignment these highly variable parts were excluded from the phylogenetic analyses. Nevertheless, careful observation revealed several sequence signatures–short stretches of sequence unique for a given clade–for some clusters of sequences. For examples, the “*L. arcyrioides*" clade has consistently shorter helices than other *Lamproderma* (e.g. helix E8_1, Fig. 4B).

### Introns

We founded introns in all nine insertion sites previously recorded for Myxomycetes [46] (Table S2). Introns had a mean length of 591 bp (maximum 1718 bp), representing up to 72% of the total sequence. Only six taxa lacked introns (*Lamproderma ovoideum*, *L. ovoideoechinulatum*, both specimens of *L. scintillans*, *Paradiacheopsis solitaria* and *Stemonitopsis hyperopta*) (Table S2). Intron sequences were very variable and were found to be similar only in sequences that had otherwise identical exons (*Meriderma carestiae* and *M. carestiae* var. *“retisporum"*, *L. echinosporum*).

## Discussion

### Characters distinguishing *Lamproderma* and allied genera

*Lamproderma* Rostaf. is well defined by a shining peridium, an usually stalked sporophore, with a solid and fibrous stalk extending into the sporotheca ( = the spore mass surrounded by the peridium); the part of the stalk inside the sporotheca, called the columella, reaches only half of the sporotheca [20] (Fig. 1D). Three genera, *Diacheopsis*, *Elaeomyxa*, and *Colloderma*, differ mainly by the absence of stalk and columella but share the shining peridium (Fig. 1 E–H). All are nested firmly within *Lamproderma* (Fig. 2). *Diacheopsis* was erected to accommodate a nivicolous species, *D. metallica*, with a peridium similar to that of *Lamproderma* but devoid of stalk and columella [47]. It now includes 16 species [20]. Because of its affinities to both *Lamproderma* and *Colloderma*, it was considered problematic and perhaps only a form of unstalked *Lamproderma* [37]. The three species of *Diacheopsis* included in this study are paired to sessile or shortly stalked *Lamproderma*: *Diacheopsis* “*cristata*" and *L. cristatum*; *D. pauxilla* and *L. zonatum* (both sequences >98% of similarities); and the *D. metallica* sequence is identical to *L. spinulosporum* (Fig. 2). None of the three *Diacheopsis* species are specifically related to each other; probably *D. metallica* could be regarded as a variety of *L. spinulosporum* and the other two could be transferred to *Lamproderma*. Perhaps all *Diacheopsis* are polyphyletic variants of *Lamproderma* that have independently lost stalks; if so a separate genus is not justified. *Elaeomyxa*, a genus of only four species, stands out in Stemonitales by its “waxy" deposits around the sporotheca. The four species of *Colloderma* are embedded, while maturing, in a layer of “slime". The strong but distant relationship of *Elaeomyxa cerifera* and *Colloderma robustum* (Fig. 2) suggests that the “wax" of *Elaeomyxa* and the “slime" of *Colloderma* are homologous. Our observations on the development of *C. robustum* and *C. oculatum* suggest that the “slime" is not a substance unknown to other Myxomycetes. (Fig. 1F). Usually, the slime layer dries out and shrinks with sporocarp development, but in the very damp environments where these two *Colloderma* species are found a generous amount is left around the maturing sporophores When dry, these remnants appear like an outer layer of the peridium and sometimes form a Physarales-like short stalk (Fig. 1G). However *Colloderma oculatum* was not related to *Elaeomyxa cerifera* but weakly though consistently associated with *Lamproderma sauteri,* making *Colloderma* polyphyletic (Fig. 2). Thus, this developmental particularity, although remarkable, appears to be too easily evolved to have much phylogenetic significance. Clearly stalk and columella have been lost repeatedly in “*Lamproderma*" (Fig. 3). The most phylogenetically significant character linking these four genera is the persistent, mostly shining peridium–all taxa possessing it that have been sequenced to date belong in the “*Lamproderma*" group of Fig. 2.

A character considered essential in delimiting myxomycete species is spore ornamentation. The “standard" *Lamproderma* and *Meriderma* spores are covered with spinules or warts, so it is noteworthy when a deviating ornamentation is found, such as reticulation or cristae (Fig. 3). Reticulation consists in somewhat aligned warts, giving the appearance of a more or less complete network on the surface of the spore (like in *L. retirugisporum*, *M. cribrarioides*). In *Meriderma*, there is a continuum from evenly spread spinules (*M. aggregatum*, *M. echinulatum*, Fig. 1I) to somewhat aligned spines (*M. spinulosporum*), better aligned ones (*M. carestiae*, more pronounced in var. *“retisporum"*, Fig. 1J), and spines forming a (nearly) complete reticulum (*M. cribrarioides*, Fig. 1K) [20]. In our study, *M. carestiae* and its yet undescribed variety “*retisporum*" had identical SSU sequences, suggesting that the slight difference in spore ornamentation may be too trivial to merit description as a new variety. *M. carestiae*, *M. cribrarioides*, and *M. aggregatum* are closely related but significantly different (>97% similarities). Unique spore cristae are found in *Lamproderma cacographicum*, which has an isolated phylogenetic position. Reticulate spores are found consistently in the *Lamproderma lycopodiicola* clade, and also distinguish the otherwise closely related *L. retirugisporum* from *L. aeneum* (“*L. aeneum*" clade, Fig. 3A and *L* *. cristatum*+*D. cristata* in the “*L. arcyrioides"* clade Fig. 3B. Taken together, these findings suggest that spore ornamentation is often a good character for distinguishing closely related taxa.

Another feature used to group several species of *Lamproderma*, the dark spots on the peridium (maculae) (Fig. 1L–O), seem to have appeared in three clades independently: *L. acanthosporum*, *L. maculatum*+*L. pseudomaculatum* and *L. echinosporum* do not cluster together in our analyses (Fig. 2). Similarly, species displaying white splinters on the peridium failed to group in a single clade (“*L. maculatum*" clade and one of the “*L. arcyrioides*" clades, Fig. 1M–N).

### The newly erected genus *Meriderma* is confirmed

This clearly monophyletic “*Meriderma*" clade is characterized by an early evanescent peridium. While drying up, it stays attached to the ends of the capillitium, which hence appear funnel-shaped [20] (Fig. 1C). *Comatricha rubens* has not been included in the genus, because the characteristic capillitium is present only at the base of the sporotheca. Our results show that it really belongs to *Meriderma*, although it is, in the current state of knowledge, the only non-nivicolous member of the clade.

### Ecological characteristics

Only two clades seemed to share unique ecological requirements: “*Stemonitis*" and “*Comatricha*" are (nearly) exclusively lignicolous (at least one exception: *Stemonitis herbatica*, fruiting on litter) (Fig. 3A). All taxa included here in the “*Stemonitis*" clade have a hollow stalk, while it is solid in *Meriderma*, *Comatricha* and *Lamproderma* [20], showing the taxonomic importance of this character. On the other hand, neither bryophilous nor nivicolous species cluster in a single clade. The nivicolous habit must have been arisen at least twice in Myxomycetes, because it is absent in the sister-group (*Ceratiomyxa fruticulosa*) and in the basal clades of bright and dark-spored Myxomycetes (*Cribraria* and Echinosteliales) [25]. Nivicolous species are found in both bright-spored (*Licea*, *Arcyria*, *Dianema*, *Trichia* and *Hemitrichia*) and dark-spored clades (*Comatricha*, *Enerthenema*, *Meriderma*, *Lamproderma*, *Diacheopsis*, *Didymium*, *Diderma*, *Lepidoderma*, *Badhamia* and *Physarum*) [20]. In the “*L. arcyrioides*" clade, all taxa except *L. violaceum* are nivicolous (Fig. 3B). This suggests that these apparently harsh climates do not represent a challenge for myxomycete species since many taxa readily adapted themselves to the nivicolous ecological conditions.

### Diverging rates of evolution between clades

A striking characteristic of the phylogenetic tree is the variation in branch lengths: specimens ascribed to the same species (e.g. *L. columbinum*, *L. scintillans*) show greater genetic distances than clades including different genera (e.g. “*L. arcyrioides*" and “*L. ovoideum*") (Fig. 2). The genetic depth within *L. columbinum* and *L. scintillans* makes it highly likely that each is an aggregate of cryptic species, as has been demonstrated for the *Didymium iridis* species complex, in which at least seven sexual biological species and 50 asexual reproducing isolates have been found [48]. The two *D. iridis* sequences included in our study also show considerable genetic distance (Fig. 2). In the case of *L. columbinum*, we could not find significant morphological differences between clades, precluding any identification by microscopy.

### Diversity of Echinosteliales

In this order, two families are currently recognized: Clastodermataceae including *Clastoderma* and the monospecific *Barbeyella* [49]; and the monogeneric Echinosteliaceae. In addition, the group includes a modified echinostelid, *Semimorula liquescens* [50]. From previous phylogenies, it was assumed that Echinosteliales lacked melanin pigments, since all spores of sequenced specimens were colourless or very faintly coloured [27]. Surprisingly, the jet-black spored *Barbeyella minutissima* is related to *Echinostelium arboreum*, implying that the (nearly) transparent spores in *Echinostelium* resulted from a secondary loss (or lack of expression) of pigments. It also suggests that Echinosteliales may be the sister-group of the dark-spored clade, as indicated by SSU trees, although this topology is not recovered in EF-1α phylogenies [25]. In spite of the colour difference, *Barbeyella minutissima* and *Echinostelium arboreum* are united by a morphological character, the peridium breaking up in peridial plates remaining attached to the capillitium.

### Conclusions

Our study highlights certain morphological characters that are relatively stable and phylogenetically informative, in this case the shining peridium to delimit the genus *Lamproderma*, the splitting-out peridium as a characteristic of the genus *Meriderma* (even when not pronounced, as in *Comatricha rubens*). On the other hand, it reveals that some characters traditionally used to delimit myxomycete genera or species can be easily acquired or lost: this is so for the stalk and columella, the nivicolous habit, the maculate peridium, and white splinters in the peridium. Despite this, we found some sound relationships between morphology and genetics. However, our study also reveals large differences in rates of evolution between clades that could blur the phylogenetic picture. Whether these differences are restricted to rDNA or are genome-wide or relate to life cycle features such as sexuality and clonality can only be established by population genetic and phylogenetic studies with multiple molecular markers. Our results indicate that the first part of the SSU rRNA gene is a valuable tool to recognize and distinguish species.

## Supporting Information

Supporting Information S1 SSU alignment in fasta format of the 84 taxa used for the tree in Fig. 2, with masks of different stringency (1404, 1490 and 1533 positions).
(FSA)

Figure S1 **A:** List of the primers used in this study and their sequences (5′-3′). Colours match the regions in the diagram of the SSU gene, showing the approximate position of the primers. Primers in bold were designed during this work. **B:** Schematic diagram of the SSU gene. Numbers indicate corresponding regions in the sequence of *Physarum polycephalum* X13160. Green bars show intron insertion positions and green labels show intron names.
(PDF)

Table S1 List of specimens used in this study, GenBank accession numbers and collection information.
(DOC)

Table S2 Length, position and number of introns found in SSU sequences.
(DOC)
