# Supplementary material for: 18S rDNA Phylogeny of Lamproderma and Allied Genera (Stemonitales, Myxomycetes, Amoebozoa)
Source: PLoS One. 2012 Apr 18;7(4):e35359. doi: 10.1371/journal.pone.0035359 (PMC3329430; doi:10.1371/journal.pone.0035359)

Supporting information S2

A: List of the primers used in this study and their sequences (5'-3'). Colours match the regions in the diagram (B), showing the approximative position of the primers. Primers in bold have been obtained during this work.

B: Schematic diagram of the SSU gene. Numbers indicate corresponding regions in the sequence of *Physarum polycephalum* X13160. Green bars show intron insertion positions and green labels show intron names.

A

	**FORWARD**
S1	AACCTGGTTGATCCTGCC
S2	TGGTTGATCCTGCCAGTAGTGT
S3	GATCCTGCCAGTAGTGTATGC
SAPhy	AATCTGCGAACGGCTCCGC
**S3Ste**	GGGCTTCTGACCTATCAACTAG
**S3Dark**	GTGCTTCTGACCTATCAACTAG
S414F	GAAGGCAGCAGGCGCGCAACG
S4Myx	AGCAGGCGCGHAACGTTCC
**718FL**	GTAATTCCAGCTCTAATAGCATACG
753F	GTTAAAACGCTCGTAGTCGGC
S11.5	GTGGTGAAATACGTTGACCC
S11atro	GACCTATGGCGAAAGCTG
**S11.7**	TGATCAAGAGCGAAAGTTAAGGG
S12m	GACGATCAGATACCGTCGTAGTC
S12'	CAGATACCGTCGTAGTCTTAAC
Ste12	CTATAAATGATACTGGCCAGG
S13	GAGTATGGTCGCAAGGCTG
S13.5	GAAACTTAAAGGAATTGACGG
S14	TTGACGGAAGGGCACACA
S14.5	TAATTTGACTCAACACGGGG
S15	TAAGTGGTGGTGCATGGTC
**S15C**	TAAGTGGTGGTGCATGGCC
S18	TCTTAGTTCGTGGAGTGG
**S18Phy**	TCTTAGTTCGTGGATTGATTTG
**S18'**	ATTCCGATAACGAGCGAGAC
**S26F**	CTTCTTAGACGTATCAGAGCC
**S44F**	GCCGATAAGGTTCTTGAAATGGG
**S46F**	AGTCATGCCCTTAGATGTTC
**S30Phy**	ACGAATGCGTCCCTGCCCT

	**REVERSE**
**SR4Dark**	TGTCCTCTAATTGTTACTCGA
**SU19R**	GACTTGTCCTCTAATTGTTACTCG
SR19	ACAAGTCTGGTGCCAGCA
718R	CGTTAAAGTTGTTGCGGTTA
753R	GGTTAAAACGCTCGTAGTCGGC
S900R	CCCGTTGATCAAGAGCGAAAG
**SR11**	GCAATTCGGTCACCCTCGAAC
**SR11T**	GCAATTCGGTCACCCACGAAC
**SR11G**	GCAATTCGGTCACCCCCGAAC
SR12	GCGAAAGTTAAGGGTTCGAAG
**SR12'**	ACCCTTAACTTTCGCTCTTGATC
SR13	GGAGTATGGTCGCAAGGCTG
**SR13U**	TTCAGYCTTGCGRCCATACTCC
**SR14**	ACTCTTTGTGTGCCCTTCC
SR14.1	CAAAGAGTGGAACCTGCGGC
SR15	GTAAGTGGTGGTGCATGG
SR15U	GGTGGTGCATGGCCGTTC
SR18	TCCGATAACGAGCGAGAC
**S43R**	ACCTGTTATTAACCCATTTCA
**SR26Ste**	CCCAGRACATCTAAGGGCAT
**S26R**	CATCACTGACCTGTTATTGACC
**SR30Ste**	ARGGCAGGGACGCATTCG
S44R	CAGTCATGCCCTTAGATGTTC
**SR50Dark**	AATCGGTAGCAGCGACGGG
**SRBE**	TACCTTGTTACGACTTCTGC
SRB	GCCTAGAGGAAGCAGAAGTCG
Rib2	GGTAATCGTAGGTGAACCTGC
RibB	GGTGAACCTGCAGAAGGATC

B

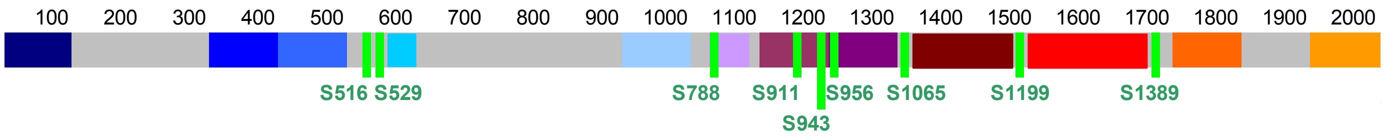

Supplement: Figure S1 — A: List of the primers used in this study and their sequences (5′-3′). Colours match the regions in the diagram of the SSU gene, showing the approximate position of the primers. Primers in bold were designed during this work. B: Schematic diagram of the SSU gene. Numbers indicate corresponding regions in the sequence of Physarum polycephalum X13160. Green bars show intron insertion positions and green labels show intron names. (PDF) [file pone.0035359.s002.pdf]
