# Supplementary material for: 18S rDNA Phylogeny of Lamproderma and Allied Genera (Stemonitales, Myxomycetes, Amoebozoa)
Source: PLoS One. 2012 Apr 18;7(4):e35359. doi: 10.1371/journal.pone.0035359 (PMC3329430; doi:10.1371/journal.pone.0035359)
Supplement: Table S1 — List of specimens used in this study, GenBank accession numbers and collection information. (DOC) [file pone.0035359.s003.doc]

**Table S1** List of specimens used in this study, GenBank accession numbers and collection information.

| **Taxon** | **Authors** | **Voucher** | **Date** | **Place of collection** | **Altitude (m)** | **Substrate** | **Lat** | **Long** | **GenBank accession #** |
| --- | --- | --- | --- | --- | --- | --- | --- | --- | --- |
| *Barbeyella minutissima* | Meylan | MM36759 | 20.10.06 | FR, Savoy, Rognaix | 1533 | Rotten log with hepatics | 45.5647°N | 06.4211°E | JQ031956 |
| *Brefeldia maxima* | (Fr.) Rostaf. | MM24519 | 07.11.04 | FR, Savoy, Esserts-Blay | 400 | Living *Populus nigra* | 45.6170°N | 06.4345°E | JQ031957 |
| *Colloderma oculatum* | (C. Lippert) G. Lister | HS2885 | 26.11.05 | FR, Haute-Savoie, Chens-le-Pont | 400 | Mosses | 46.2844°N | 06.2826°E | JQ031959 |
| *Colloderma robustum* | Meylan | AMFD270 | 30.09.07 | DE, Saxony, Pirna, Utterwalde | 249 | Bolder, *Mylia taylorii* | 50.977°N | 14.039°E | JQ031960 |
| *Comatricha anastomosans* | Kowalski | Now12905 | 09.06.04 | AT, Ebensee, Feuerkogel | 1500 | Dead *Pinus mugo* | 47.8141°N | 13.7188°E | JQ031961 |
| *Comatricha anastomosans ** | Kowalski | Now11379 | 25.05.01 | AT, Ebensee, Feuerkogel | 1600 | Dead *Pinus mugo* | 47.8141°N | 13.7188°E | JQ031962 |
| *Comatricha pseudoalpina ** | G. Moreno, H. Singer, A. Sánchez & Illana | MM32556 | 20.05.01 | FR, Savoy, Bonneval-Les Monts | 1537 | *Vaccinum myrtillus* | 45.5339° N | 06.4434° E | JQ031963 |
| *Comatricha rubens* | Lister | MM29181 | 16.12.03 | FR, Corse, Alata | 100 | *Pinus pinea* and *Quercus suber* litter | 41.9763°N | 08.7425°E | JQ031958 |
| *Diachea subsessilis* | Peck | MM24463 | 28.98.04 | FR, Savoy, Saint-Paul-sur-Isère | 391 | *Urtica* | 45.5919°N | 06.4447°E | JQ031964 |
| *Diacheopsis "cristata" ** | ad int. | MM38009 | 19.03.09 | FR, Savoy, Doucy, Le Cretet | 1080 | Litter of *Fagus sylvatica* | 45.5135°N | 06.4696°E | JQ031965 |
| *Diacheopsis pauxilla* | Mar. Meyer & Poulain | MM29883 | 14.06.06 | FR, Savoy, La Bâthie | 1804 | *Vaccinum myrtillus* | 45.6503°N | 06.4794°E | JQ031966 |
| *Elaeomyxa cerifera* | (G.Lister) Hagelst. | MM24498 | 30.09.04 | FR, Savoy, Rognaix, Le Laitelet | 1700 | Picea stump | 45.5597°N | 06.4175°E | JQ031967 |
| *Lamproderma acanthosporum* | Kowalski | MM36058 | 02.05.06 | FR, Savoy, Esserts-Blay | 1397 | *Vaccinium myrtillus* | 45.6264°N | 06.4045°E | JQ031968 |
| *Lamproderma aeneum* | Mar. Meyer & Poulain | MM36255 | 16.05.06 | FR, Savoy, Hautecour | 1630 | *Rubus* | 45.5211°N | 06.5413°E | JQ031969 |
| *Lamproderma aeneum ** | Mar. Meyer & Poulain | AK06013 | 19.04.06 | JP, Hokkaido, Sapporo | 25 | Dead stem on soil | 43.0825°N | 141.3381°E | JQ031970 |
| *Lamproderma album* | H. Neubert, Nowotny & K. Baumann | MM37151 | 24.05.07 | FR, Savoy, La Bâthie | 1798 | Dry stems of *Cicerbita* | 45.6504°N | 06.4795°E | JQ031971 |
| *Lamproderma album ** | H. Neubert, Nowotny & K. Baumann | MM35162 | 26.05.05 | FR, Savoy, Monthion | 1659 | *Picea* dead branches | 45.6219°N | 06.3922°E | JQ031972 |
| *Lamproderma arcyrioides* | (Sommerf.) Rostaf. | MM37005 | 22.04.07 | FR, Savoy, Beaufort, Col Méraillet | 1600 | *Vaccinum myrtillus* | 45.6938°N | 06.6317°E | JQ031973 |
| *Lamproderma arcyrionema #* | Rostaf. | MM27880 | 22.08.01 | FR, Savoy, St-Paul-sur-Isère | 400 | Decayed log | 45.5919°N | 06.4447°E | JQ031975 |
| *Lamproderma cacographicum* | Bozonnet, Mar. Meyer & Poulain | AMFD310 | 28.04.08 | FR, Savoy, Méribel-Mottaret | 1687 | Dead trunk | 45.3712°N | 06.5803°E | JQ031976 |
| Lamproderma cf. arcyrioides * | (Sommerf.) Rostaf. | AMFD338 | 29.05.08 | JP, Hokkaido, Uruy forest, site 1, U07 | 595 | Living leaves of *Sasa kurilensis* | 44.4330°N | 142.1479°E | JQ031974 |
| *Lamproderma cristatum* | Meylan | MM37003 | 22.04.07 | FR, Savoy, Col Méraillet | 1600 | *Vaccinium myrtillus* | 45.6938°N | 06.6317°E | JQ031977 |
| *Lamproderma disseminatum #* | Kowalski | AMFD38 | 03.06.00 | FR, Savoy, La Plagne | 2000 | Decorticated log of *Pinus cembra* | 45.5078°N | 06.6823°E | JQ031978 |
| *Lamproderma echinosporum* | Meylan | AK06016 | 19.04.06 | JP, Hokkaido, Sapporo | 25 | Dead stem on soil | 43.0819°N | 141.3393°E | JQ031979 |
| *Lamproderma echinosporum* | Meylan | AMFD136 | 01.05.04 | CH, Glaris, Näfels, Fronalp | 1360 | Dead twigs on soil | 47.078°N | 9.1128°E | JQ031980 |
| *Lamproderma lycopodiicola* | Kuhnt | AMFD309 | 06.05.08 | GB, Scotland, Cairngorm, Choire Chais | 884 | *Lycopodium annotinum* | 57.1226°N | 3.6628°W | JQ031981 |
| *Lamproderma maculatum* | Kowalski | MM37059 | 13.05.07 | FR, Savoy, La Bâthie | 1850 | *Rubus idaeus* | 45.6490°N | 06.4791°E | JQ031982 |
| *Lamproderma ovoideoechinulatum* | Mar. Meyer & Poulain | JMF527 | 27.05.02 | JP, Yamanashi, Mt. Fuji | 2500 | Fallen twigs near snow | n.a. | n.a. | JQ031983 |
| *Lamproderma ovoideum* | Meylan | AK06022 | 25.04.06 | JP, Hokkaido University Campus | 25 | Dead stem on soil | 43.0836°N | 141.3391°E | JQ031984 |
| *Lamproderma pseudomaculatum* | Mar. Meyer & Poulain | MM37354 | 09.03.08 | FR, Savoy, La Bathie | 1366 | Dead twigs under *Populus tremula* | 45.6296°N | 06.4746°E | JQ031985 |
| *Lamproderma pseudomaculatum ** | Mar. Meyer & Poulain | AMFD180 | 19.03.03 | FR, Savoy, Hautecour | 1400 | Litter of *Populus* leaves | 45.5218°N | 06.5477°E | JQ031986 |
| *Lamproderma pulchellum* | Meylan | MM36096 | 04.05.06 | FR, Hautes-Alpes, Le Casset | 1525 | Living bush | 44.9853°N | 06.4795°E | JQ031987 |
| *Lamproderma pulveratum* | Mar. Meyer & Poulain | MM37016 | 29.04.07 | FR, Savoy, Esserts-Blay | 1555 | *Picea* branches on soil | 45.6174°N | 06.4000°E | JQ031988 |
| *Lamproderma retirugisporum* | G. Moreno, H. Singer, Illana & A. Sánchez | MM23831 | 08.05.04 | IT, Cuneo, Bagni di Vinadio | 1260 | *Rubus* dry branches | 44.2876°N | 07.0775°E | JQ031989 |
| *Lamproderma retirugisporum ** | G. Moreno, H. Singer, Illana & A. Sánchez | MM32478 | 29.04.10 | FR, Savoy, Courchevel | 1750 | Dead grass on soil | 45.4053°N | 06.6626°E | JQ031990 |
| *Lamproderma sauteri* var. *pulchrum ** | Meylan | AMFD336 | 29.05.08 | JP, Hokkaido, Uruy forest | 595 | Living twigs | 44.4330°N | 142.1479°E | JQ031991 |
| *Lamproderma scintillans* | (Berk. & Broome) Morgan | MA70223 | 09.12.06 | ES, Valencia, Onteniente | 350 | *Cercis siliquastrum* dead leaves | 38.81°N | 06.17°W | JQ031993 |
| *Lamproderma scintillans* | (Berk. & Broome) Morgan | JM3204 | 15.04.01 | JP, Tokyo, Okutama-cho | 700 | Dead leaf | n.a. | n.a. | JQ031992 |
| *Lamproderma* sp. nov. *"carpatiensis"* |  | MM UK14 | 28.01.09 | UA, Gimba, Pilipets | 1025 | *Lycopodium* sp. | 48.6464°N | 23.2541°E | JQ031994 |
| *Lamproderma* sp. nov. "cf. *muscorum"* |  | MM37253 | 07.09.07 | FR, Savoy, Rognaix | 864 | Mosses on North exposed slope | 45.5791°N | 06.4248°E | JQ031995 |
| *Lamproderma spinulosporum ** | Mar.Mey., Nowotny & Poulain | MM32506 | 20.05.01 | FR, Savoy, Bonneval-Les Monts | 1537 | Living *Vaccinum myrtillus* | 45.5339°N | 06.4434°E | JQ031996 |
| *Lamproderma violaceum ** | Fr. ex Rostaf. | MM29783 | 07.11.05 | IT, Cuneo, Castel del Piano | 700 | Living *Castanea* trunk | 42.8920°N | 11.5381°E | JQ031997 |
| *Lepidoderma alpestroides ** | Mar.Mey. & Poulain | AMFD340 | 29.05.08 | JP, Hokkaido, Uryu Forest | 595 | *Sasa kurilensis* living leaves | 44.4330° N | 142.1479°E | JQ031998 |
| *Meriderma carestiae* | (Ces. & De Not.) Mar.Mey. & Poulain | MM35985 | 14.04.06 | FR. Savoy, Bonneval-Eglise | 1218 | Dead branches on soil | 45.5278°N | 06.4533°E | JQ031999 |
| *Meriderma cribrarioides* | (Fr.) Mar.Mer. & Poulain | MM37106 | 24.05.07 | FR. Savoy, La Bathie | 1798 | Living *Vaccinum myrtillus* | 45.6504°N | 06.4795°E | JQ032000 |
| *Paradiacheopsis solitaria* | (Nann.-Bremek.) Nann.-Bremek. | DM7368 | 05.10.08 | GB, East Kent, Faversham | 36 | Living *Quercus robur* bark | 51.3027°N | 00.8317°E | JQ032001 |
| *Stemonitopsis hyperopta #* | (Meyl.) Nann.-Bremek. | MM37295 | 13.10.07 | FR. Savoy, Albertville | 896 | Rotten coniferous log | 45.6805°N | 06.4204°E | JQ032002 |
| *Stemonitopsis typhina #* | (F.H.Wigg.) Nann.-Bremek. | MM36735 | 21.09.06 | FR, Alpes-Maritimes, Ile Ste-Marguerite | 20 | Decayed log of *Pinus* | 43.5153°N | 07.0555°E | JQ032003 |
| *Stemonitopsis typhina *#* | (F.H.Wigg.) Nann.-Bremek. | MM36830 | 30.09.06 | FR. Savoy, St-Paul-sur-Isère | 386 | Stump | 45.5917°N | 06.4472°E | JQ032004 |
| **Updated seqences** |  |  |  |  |  |  |  |  |  |
| Meriderma carestiae var. "retisporum" | Meylan | AMFD173 | 08.05.03 | FR. Savoy, Méribel altiport | 1850 | *Salix* branches | 46.26° N | 06.39° E | DQ903671.1 |
| Comatricha pseudoalpina | G. Moreno, H. Singer, A. Sánchez & Illana | MM23892 | 16.05.04 | FR. Savoy, La Bathie | 1614 | Decorticated branch of *Picea abies* | 45.65°N | 06.48°E | DQ903673.2 |

Legend: * = partial sequence (ca. 600 bp at the beginning of the SSU); # = sequence not included in the phylogenetic analyses. Recent synonyms*: L. arcyrioides* was *L. splendens*; *Physarum nivale* was *Badhamia panicea* var. *nivalis*; *Meriderma* *carestiae* var. "*retisporum*" was *Lamproderma* *atrosporum* var. *retisporum*; *Meriderma* *aggregatum* was *Lamproderma* *aggregatum*; *M. fuscatum* was *L. fuscatum*. Herbaria: MM=M. Meyer, France; AMFD=A. M. Fiore-Donno, Germany; HS= H. Seraoui, France; Now= W. Nowotny, Austria; AK= A. Kamono, Japan; JMF=J. Matsumoto, Fukui Botanical Garden, Japan; MA= Herbario Jardin Botanico Madrid; DM= D. Mitchell, UK.
