# Supplementary material for: 18S rDNA Phylogeny of Lamproderma and Allied Genera (Stemonitales, Myxomycetes, Amoebozoa)
Source: PLoS One. 2012 Apr 18;7(4):e35359. doi: 10.1371/journal.pone.0035359 (PMC3329430; doi:10.1371/journal.pone.0035359)
Supplement: Table S2 — Length, position and number of introns found in SSU sequences. (DOC) [file pone.0035359.s004.doc]

**Table S2**: Length, position and number of introns found in SSU sequences.

|  | **Introns names and length (nucleotides)** | | | | | |  |  |  | **Total length:** | |  | **Introns** | |
| --- | --- | --- | --- | --- | --- | --- | --- | --- | --- | --- | --- | --- | --- | --- |
| **Taxon** | **S516** | **S529** | **S788** | **S911** | **S943** | **S956** | **S1065** | **S1199** | **S1389** | **exons** | **introns** | **total** | **#** | **%** |
| *Barbeyella minutissima* |  |  |  | 581 |  |  |  |  |  | 2133 | 581 | 2714 | 1 | 21.4% |
| *Brefeldia maxima* |  |  | 503 | 642 |  |  |  |  | 1304 | 1931 | 2449 | 4380 | 3 | 55.9% |
| *Colloderma oculatum* | 719 |  | 482 |  |  | 493 | 481 | 340 | 421 | 1864 | 2936 | 4800 | 6 | 61.2% |
| *Colloderma robustum* | 509 | 842 |  | 461 | 377 | 382 | 1718 | 347 | 384 | 1954 | 5020 | 6974 | 8 | 72.0% |
| *Comatricha anastomosans* | 1411 |  | 700 | 507 | 436 | 331 | 727 |  | cDNA | 1684 | 4112 | 5796 | 6 | n.a. |
| *Comatricha pseudoalpina* | 1471 |  | 704 | 534 | 437 | 338 |  |  | 815 | 1918 | 4299 | 6217 | 6 | 69.1% |
| *Comatricha rubens* |  | 594 |  | 494 |  |  | 410 |  |  | 1897 | 1498 | 3395 | 3 | 44.1% |
| *Diachea subsessilis* |  |  | 515 | 1219 |  | 1245 |  |  | 919 | 1902 | 3898 | 5800 | 4 | 67.2% |
| *Diacheopsis pauxilla* | 516 |  | 428 | 596 |  | 398 | 457 | 432 | 628 | 1885 | 3455 | 5340 | 7 | 64.7% |
| *Elaeomyxa cerifera* | 725 |  |  | 571 |  | 1454 |  |  |  | 2007 | 2750 | 4757 | 3 | 57.8% |
| *Lamproderma acanthosporum* | 536 |  | 414 |  | 376 |  | 456 | 391 |  | 1902 | 2173 | 4075 | 5 | 53.3% |
| *Lamproderma aeneum* | 497 | 607 | 487 | 501 | 384 | 404 | 441 |  | 545 | 1896 | 3866 | 5762 | **8** | 67.1% |
| *Lamproderma album* | 993 | 584 | 1250 | 466 |  |  | 1094 | 342 |  | 1880 | 4729 | 6609 | 6 | 71.6% |
| *Lamproderma arcyrioides* |  |  | 724 | 724 |  | 445 | 642 | 418 | 1006 | 1891 | 3959 | 5850 | 6 | 67.7% |
| *Lamproderma arcyrionema* |  | 375 |  |  |  |  |  |  |  | 2339 | 375 | 2714 | 1 | 13.8% |
| *Lamproderma cacographicum* |  | 519 |  |  | 444 | 607 | 495 | 442 | 957 | 1903 | 3464 | 5367 | 6 | 64.5% |
| *Lamproderma cristatum* | cDNA | cDNA | cDNA | cDNA | cDNA | cDNA | cDNA | cDNA | cDNA | 1925 | n.a. | 1925 | n.a. | n.a. |
| *Lamproderma disseminatum* |  |  | 410 | 633 | 338 | 652 |  |  | 498 | 2171 | 2531 | 4702 | 5 | 53.8% |
| *Lamproderma echinosporum* AK06016 | 510 | 511 |  | 404 | 420 |  |  | 356 | 486 | 1894 | 2687 | 4581 | 6 | 58.7% |
| *Lamproderma echinosporum* AMFD136 | 509 | 507 | 370 | 404 |  |  |  |  | 481 | 1998 | 2271 | 4269 | 5 | 53.2% |
| *Lamproderma lycopodiicola* |  |  |  |  | 375 |  |  |  |  | 1960 | 375 | 2335 | 1 | 16.1% |
| *Lamproderma maculatum* |  | 664 | 424 |  |  | 479 |  |  |  | 1894 | 1567 | 3461 | 3 | 45.3% |
| *Lamproderma ovoideoechinulatum* |  |  |  |  |  |  |  |  |  | 1945 | 0 | 1945 | 0 | 0% |
| *Lamproderma ovoideum* |  |  |  |  |  |  |  |  |  | 1942 | 0 | 1942 | 0 | 0% |
| *Lamproderma pseudomaculatum* AMFD180 * |  | 524 | n.a. | n.a. | n.a. | n.a. | n.a. | n.a. | n.a. | 1069 | 524 | 1593 | 1 | n.a. |
| *Lamproderma pseudomaculatum* MM37354 | 1082 |  |  |  | 376 |  |  |  | 411 | 1904 | 1869 | 3773 | 3 | 49.5% |
| *Lamproderma pulchellum* |  | 596 |  | 434 |  | 675 |  |  |  | 1915 | 1705 | 3620 | 3 | 47.1% |
| *Lamproderma pulveratum* | cDNA | cDNA | cDNA | cDNA | cDNA | cDNA | cDNA | cDNA | cDNA | 1927 | n.a. | 1927 | n.a. | n.a. |
| *Lamproderma retirugisporum* |  | 659 | 469 |  |  | 469 |  |  |  | 1921 | 1597 | 3518 | 3 | 45.4% |
| *Lamproderma scintillans* JM3204 |  |  |  |  |  |  |  |  |  | 1880 | 0 | 1880 | 0 | 0% |
| *Lamproderma scintillans* MA70223 |  |  |  |  |  |  |  |  |  | 1883 | 0 | 1883 | 0 | 0% |
| *Lamproderma* sp. nov. *"carpatiensis"* | 449 |  |  |  |  |  |  | 324 |  | 1865 | 773 | 2638 | 2 | 29.3% |
| *Lamproderma* sp. nov. "cf*. muscorum"* |  | 704 |  |  | 852 |  |  | cDNA | cDNA | 1992 | 1556 | 3548 | 2 | n.a. |
| *Meriderma carestiae* |  | 492 | 510 | 422 |  |  | 457 |  | 566 | 1882 | 2447 | 4329 | 5 | 56.5% |
| *Meriderma carestiae* var. *retisporum* |  | 492 | 510 | 425 |  |  | 457 |  | 566 | 1883 | 2450 | 4333 | 5 | 56.5% |
| *Meriderma cribrarioides* | 489 | 1432 | 474 | 393 | 449 | 466 | 429 |  | 538 | 1876 | 4670 | 6546 | **8** | 71.3% |
| *Paradiacheopsis solitaria* |  |  |  |  |  |  |  |  |  | 2232 | 0 | 2232 | 0 | 0% |
| *Stemonitopsis hyperopta* |  |  |  |  |  |  |  |  |  | 2292 | 0 | 2292 | 0 | 0% |
| *Stemonitopsis typhina* |  | 1587 | 354 |  |  | 1363 |  |  |  | 2157 | 3304 | 5461 | 3 | 60.5% |
| **Mean:** |  |  |  |  |  |  |  |  |  | **1933** | **2159** | **3982** | **4** | **44%** |
| **Number of introns:** | **14** | **17** | **18** | **19** | **12** | **16** | **13** | **9** | **16** |  |  |  |  |  |
